# REgistry-based randomized controlled trial of treatment and Duration and mortality in long-term OXygen therapy (REDOX) study protocol

**DOI:** 10.1186/s12890-019-0809-7

**Published:** 2019-02-26

**Authors:** Josefin Sundh, Anna Bornefalk-Hermansson, Zainab Ahmadi, Anders Blomberg, Christer Janson, David C. Currow, Christine F. McDonald, Nikki McCaffrey, Magnus Ekström

**Affiliations:** 1 Department of Respiratory Medicine, School of Medical Sciences, Örebro University, Örebro, Sweden; 2 UCR Uppsala Clinical Research Center, Uppsala University, Uppsala, Sweden; 3 Department of Clinical Sciences, Division of Respiratory Medicine & Allergology, Lund University, Lund, Sweden; 4 Department of Public Health and Clinical Medicine, Division of Medicine/Respiratory Medicine, Umeå University, Umeå, Sweden; 5 Department of Medical Sciences, Respiratory, Allergy & Sleep Research, Uppsala University, Uppsala, Sweden; 6 Faculty of Health, University of Technology, Sydney, Australia; 7 Institute for Breathing and Sleep, Melbourne, Victoria Australia; 8 Deakin Health Economics, Deakin University, Burwood, Victoria Australia

**Keywords:** Register-based randomized controlled trial, Hypoxaemia, Long-term oxygen therapy, Oxygen duration, Chronic obstructive pulmonary disease, Interstitial lung disease, Mortality, Hospitalizations, Health-related quality of life, Symptoms

## Abstract

**Objective:**

Long-term oxygen therapy (LTOT) during 15 h/day or more prolongs survival in patients with chronic obstructive pulmonary disease (COPD) and severe hypoxemia. No randomized controlled trial has evaluated the net effects (benefits or harms) from LTOT 24 h/day compared with 15 h/day or the effect in conditions other than COPD. We describe a multicenter, national, phase IV, non-superiority, registry-based, randomized controlled trial (R-RCT) of LTOT prescribed 24 h/day compared with 15 h/day. The primary endpoint is all-cause-mortality at 1 year. Secondary endpoints include cause-specific mortality, hospitalizations, health-related quality of life, symptoms, and outcomes in interstitial lung disease.

**Methods/design:**

Patients qualifying for LTOT are randomized to LTOT 24 h/day versus 15 h/day during 12 months using the Swedish Register for Respiratory Failure (Swedevox). Planned sample size in this pragmatic study is 2126 randomized patients. Clinical follow-up and concurrent treatments are according to routine clinical practice. Mortality, hospitalizations, and incident diseases are assessed using national Swedish registries with expected complete follow-up. Patient-reported outcomes are assessed using postal questionnaire at 3 and 12 months.

**Discussion:**

The R-RCT approach combines the advantages of a prospective randomized trial and large clinical national registries for enrollment, allocation, and data collection, with the aim of improving the evidence-based use of LTOT.

**Trial registration:**

Clinical Trial registered with www.clinicaltrials.gov, Title: REgistry-based Treatment Duration and Mortality in Long-term OXygen Therapy (REDOX); ID: NCT03441204.

## Background

Long-Term Oxygen Therapy (LTOT) is an established treatment to improve survival in patients with chronic daytime hypoxemia due to chronic obstructive pulmonary disease (COPD) [1]. Internationally accepted guidelines recommending that LTOT should be given for 15 h/day or more [2] are based on two small randomized controlled trials (RCTs) conducted in the 1970s with a total of 290 patients; the ‘Continuous or Nocturnal Oxygen Therapy’ trial (NOTT) published in 1980 [3] and the ‘Medical Research Council’ (MRC) study published in 1981 [4]. In NOTT, prescribed oxygen duration 24 h/day (mean use 18 h/day) increased survival compared with 12 h/day, and in the MRC study oxygen 15 h/day increased survival compared with no oxygen. The internationally accepted recommendation to use LTOT at least 15 h/day and preferably 24 h/day is based on an unadjusted observational comparison of the treatment arms of these two trials, where the crude mortality rate was lower for the 18 h/day group in NOTT than for the 15 h/day group in the MRC trial [5] (21% vs 41%). However, the presumed survival benefit of continuous LTOT over 15 h /day has not been proven in a randomized controlled trial. Our recently performed observational study of 2249 patients using LTOT for hypoxemic COPD suggested that there may indeed be no differences in survival for LTOT 24 h/day compared with 15 h/day [6]. As for LTOT to patients with mild hypoxemia at rest or with isolated hypoxemia during exertion, the recent ‘Long Term Oxygen Therapy’ (LOTT) trial showed no reduced mortality [7], and a Cochrane metaanalysis reported some effect on breathlessness but not on health related quality of life (HRQoL) [8].

LTOT is associated with considerable logistic challenges, costs and side effects [9, 10]. Being connected to oxygen equipment is associated with feelings of shame, restrictions of daily activities, and social isolation [11, 12]. Thus, continuous LTOT may pose an unnecessary burden and limitation for many patients compared with treatment 15 h/day, where they can be free of their oxygen for 9 h/day. The longer the duration of daily therapy, the less likelihood of adherence to treatment. Low-flow oxygen therapy has been associated with oxidative stress and inflammation [13], which could potentially contribute to increased morbidity and negative health effects [14, 15]. Moreover, the validity of the NOTT and MRC trials for current practice is questionable as the studies included mainly men and people younger than 70 years without significant comorbidity. Of note, treatment for COPD and cardiovascular disease has also improved in recent decades [5, 9]. The results of the NOTT and MRC trials are also generally applied for all conditions with secondary hypoxemic respiratory failure, although intervention studies of LTOT in pulmonary fibrosis, heart failure and pulmonary hypertension are lacking. Thus, improved evidence-based LTOT is needed.

The Registry-based randomized controlled trial (R-RCT) is a recent scientific paradigm shift that can facilitate large interventional trials with adequate power and low cost [16] to detect clinically important patient outcomes pragmatically in the real world setting [17]. The trial design was pioneered by Swedish cardiologists in the TASTE study of thrombus aspiration using the Swedish Coronary Angiography and Angioplasty Registry, which enabled inclusion of a large nationally representative sample, and the study was highly cost-effective [16].

This paper presents the protocol for a Swedish national multisite R-RCT; REgistry based randomized controlled trial of treatment Duration and mortality in long-term OXygen therapy (REDOX), to test the primary research hypothesis that LTOT 24 h/day does not reduce all-cause mortality compared with LTOT 15 h/day in patients with oxygen-dependent COPD. Secondary outcomes include cause-specific mortality, hospitalizations, health-related quality of life, symptoms, effects in other underlying diseases, and effects by the level of hypoxemia at baseline.

## Methods/design

### Objectives

The main aim is to determine whether oxygen prescribed continuously 24 h/day changes all-cause-mortality rate at 1 year in a similar way as oxygen prescribed for 15 h/day during long-term oxygen therapy (LTOT). Secondary outcomes include mortality rate from all causes at 3 and 12 months, mortality rate from respiratory disease at 3 and 12 months, mortality rate from cardiovascular disease at 3 and 12 months, hospitalization rate from all causes at 3 and 12 months, hospitalization rate with a primary diagnosis of respiratory disease or respiratory infection at 3 and 12 months, hospitalization rate with a primary diagnosis of cardiovascular disease at 3 and 12 months, rate of an incident diagnosis of cardiovascular disease at 3 and 12 months, self-rated oxygen utilization, breathlessness, fatigue, informant-rated cognition, self-rated cognition, health-related quality of life (HRQoL), global impression of change from baseline, self-reported physical activity and preference of continuing treatment. All key research questions are bulleted in Table 1.

**Table 1:** Key research questions addressed by this study protocol

Does LTOT prescribed for 24 h/day versus 15 h/day:	
Primary:	
• Fail to reduce mortality rate from all causes?	
Secondary:	
• Fail to reduce mortality rate from respiratory disease?	
• Fail to reduce mortality rate from cardiovascular disease?	
• Fail to reduce hospitalization rate from all causes?	
• Fail to reduce hospitalization rate from respiratory disease?	
• Fail to reduce hospitalization rate from cardiovascular disease?	
• Fail to reduce the rate of an incident diagnosis of cardiovascular disease?	
• Fail to reduce the level of breathlessness?	
• Fail to reduce fatigue?	
• Fail to improve level of self-reported physical activity?	
• Fail to improve health-related quality of life?	
• Fail to improve cognition?	
• Fail to decrease the rate of LTOT withdrawal?	
• Fail to increase the patient’s preference in continuing LTOT?	

Abbreviations: LTOT = Long-term oxygen therapy

### Design and participants

**Fig. 1:**
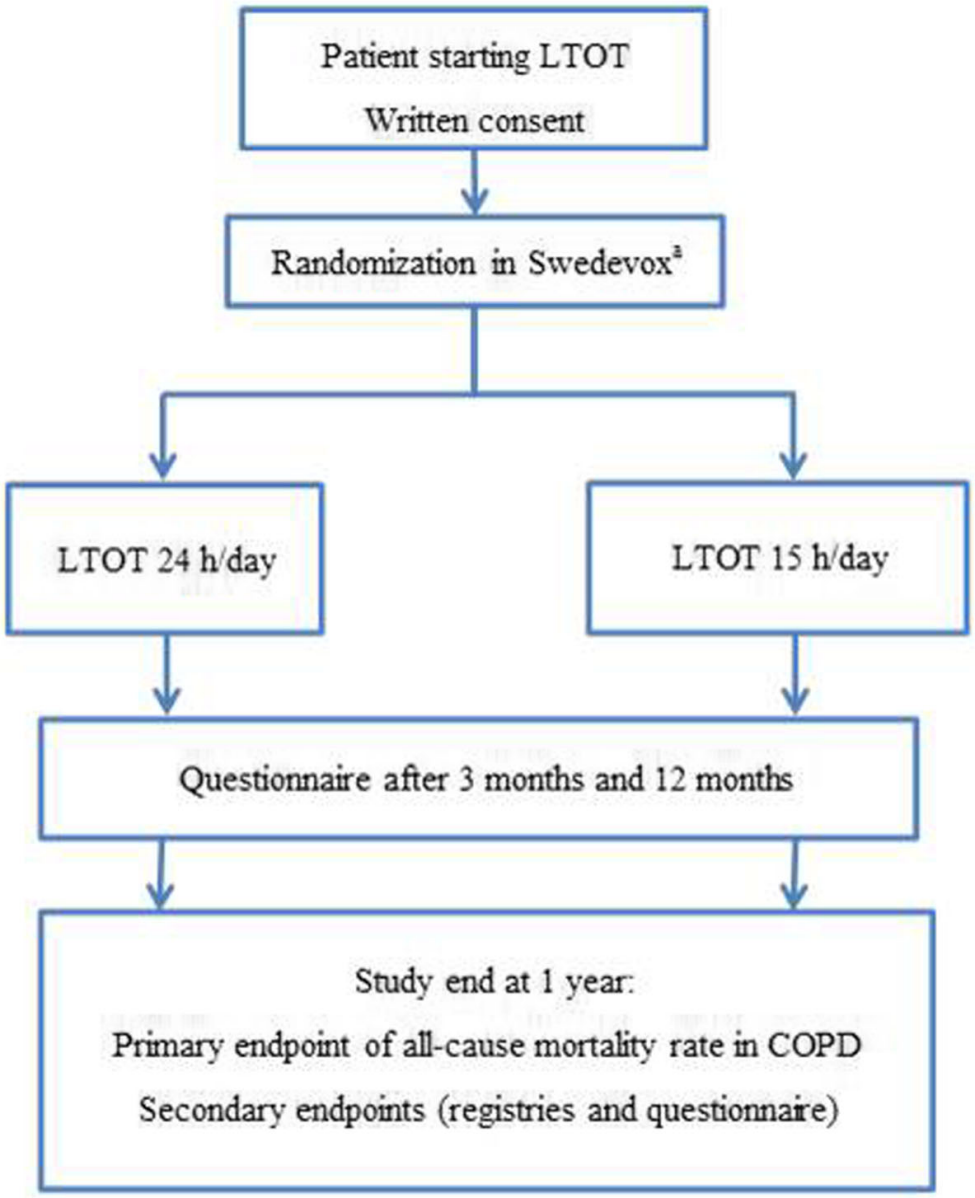
Flow chart of the outline of the study. ^a^Randomization at or within four weeks of starting LTOT. *Abbreviations:* LTOT = Long-term oxygen therapy. COPD = Chronic Obstructive Pulmonary Disease

The REDOX is a multicenter, single-blinded (analyst), effectiveness, phase IV R-RCT. Current Swedish guidelines recommend LTOT to be used for at least 15–16 h/day but preferably 24 h/day [18]. In the present study, patients starting LTOT will be randomized to either oxygen prescribed 24 h/day or 15 h/day using the Swedish Register for Respiratory Failure (Swedevox). Clinical follow-up and concurrent treatments are according to routine clinical practice. The main endpoints of mortality, hospitalizations, and incident disease are assessed using Swedish registry data, with expected complete follow-up due to compulsory registrations nationally. Patient-reported outcomes are assessed using postal questionnaire at three and 12 months. The study is managed by the Uppsala Clinical Research Center (UCR), a clinical trial unit with great experience from national randomized controlled trials. All 48 centers prescribing LTOT in the Swedish Quality Register for Respiratory Failure (Swedevox) are invited to participate in the trial. The study design is outlined in Fig. 1. The inclusion and exclusion criteria for the trial are listed in Table 2. As all patients eligible for starting LTOT are included regardless of the underlying disease, the overall study population is expected to be heterogeneous. The main analysis will be in the defined group of patients with spirometrically verified COPD and severe hypoxemia. Secondary analyses will also be performed in sub populations listed in Table 3.

**Table 2:** Inclusion and exclusion criteria

Inclusion Criteria	
• Age 18 years or older	
• Standard eligibility criteria for non-palliative LTOT at rest: [1, 2]	
o PaO2 < 7.4 kPa or oxygen saturation < 88% breathing air, *or* o PaO2 < 8.0 kPa on air and either signs of heart failure or polycythemia (EVF > 0.54)	
Exclusion Criteria	
• Standard contraindications for LTOT	
• Smoking or contact with open fire	
• Other inability to safely comply with LTOT	
• Already on LTOT for more than 4 weeks	
• Inability to comply with any of the study interventions (LTOT 15 h /day or 24 h/ day) as judged by the responsible oxygen staff	
• Opt out from being registered in Swedevox	
• Inability to give informed written consent to participate in the study as judged by the responsible oxygen staff	
• Lack of Swedish identification number	
• Previous participation in the study	

Abbreviations: *LTOT* Long-term oxygen therapy, *PaO*_*2*_ arterial blood gas tension of oxygen, *EVF* Erythrocyte Volume Fraction

**Table 3:** Analysis populations

Primary analysis population:	
I. Participants with verified COPD (FEV_1_/FVC < 0.7 after bronchodilation) with severe resting hypoxemia (PaO_2_ < 7.4 kPa) when starting LTOT	
Secondary analysis populations:	
II. Participants with ILD as main reason for starting LTOT with severe hypoxemia (PaO_2_ < 7.4 kPa breathing air) at LTOT start	
III. Participants with moderate hypoxemia (PaO2 7.4–8.0 kPa on air) at LTOT start by diagnosis and as a discrete group	
IV. All participants	

Abbreviations: *COPD* Chronic Obstructive Pulmonary Disease, *FEV*_*1*_ Forced Expiratory Volume in one second, *FVC* Forced Expiratory Volume, *PaO*_*2*_ arterial blood gas tension of oxygen, *LTOT* Long-term oxygen therapy, *ILD* interstitial lung disease

### Recruitment

All units prescribing and managing LTOT in Sweden report to Swedevox registry, which has a nationwide coverage of 85% of patients starting LTOT [18]. Approximately 70% of patients start LTOT due to COPD and 15% due to interstitial lung disease (ILD) [3]. In accordance with Swedish legislation and regulations, patients starting LTOT are given written information about Swedevox including the choice to opt-out from registration in Swedevox or for their data to be removed from the register at any time.

The oxygen staff at all units will be instructed to register all patients eligible for LTOT in Swedevox, using the standard online interface at the time of starting the oxygen treatment, in accordance with current clinical routine. The patients will be informed about the study verbally and by written information, that taking part is entirely voluntary and that they can withdraw from study whenever they want without this affecting their usual care. Written informed consent will be obtained from all participants before randomization. Participants will be eligible for inclusion up to 4 weeks after the start date of LTOT.

Before randomization, the patient’s Swedish identity number; start date; and type of oxygen therapy (i.e. LTOT and not palliative or ambulatory oxygen therapy) will be entered in Swedevox. Eligible patients are identified and written informed consents or reasons for non-participation will be documented in a study specific module of the Swedevox registry. To facilitate randomization, the Swedevox registry will have a randomization list prepared by a study independent statistician. Patients will be randomized in a 1:1 ratio to be prescribed either LTOT 24 h/day or 15 h/day. Swedevox will directly show which LTOT duration the patient should receive and the patient will be given standardized written information about the allocated treatment. The first study day is defined as the day of randomization. Patients can only enter the study once. The staff will inform the patient and caregiver about the allocated daily oxygen duration in the patient’s medical record (as part of routine care) to facilitate subsequent follow-up of the oxygen use. National guidelines recommend that ambulatory and active patients are prescribed portable oxygen equipment to facilitate adherence to the prescribed daily oxygen duration [18].

This is a single-blinded (analyst) trial. The patient, principal investigators and co-investigators, responsible study nurses, study monitor, caregiver, and the clinical oxygen staff will be aware of the allocated daily treatment duration. The patient and caregiver will receive standardized written information about the study and the allocated LTOT duration. The patient and caregiver will be instructed on the importance of using the LTOT as prescribed and to inform the oxygen staff about any changes in oxygen use which are documented in the patient’s medical record. The UCR study staff and statistician responsible for analyzing the study data will be blinded to the allocated treatment group. The objective primary endpoint of all-cause mortality in a single blinded trial design is robust and minimizes the risk of ascertainment and reporting bias [19].

### Power and sample size

Based on Swedevox data which have been stable over the last 5 years [18], the one year survival probability with LTOT prescribed 15 h/day is 0.70. For superiority, the survival probability should exceed 0.74 for LTOT 24 h/day. The clinical limit of superiority is thus an increase in survival of 6%. With a non-superiority test for two survival curves using a Cox proportional hazards model, significance level 0.05, power 80%, balanced allocation, the assumptions of a constant hazard ratio throughout the study and complete follow-up of subjects, the estimated sample size is 1063 patients. Allowing for that up to 50% of patients in Swedevox may not be included in the primary intention to treat (ITT) analysis set due to non-COPD, or no verified severe hypoxemia (based on current Swedevox data). The final planned total trial size is 2126 randomized patients. Given that 1300 patients start LTOT in Swedevox each year (which has been stable over the last 5 years), of whom approximately 850 have COPD, and a rate of recruitment to the study of 75%, study recruitment is estimated to be completed within 27 months.

### Measurements

The pre-specified endpoint measurements include registry-based outcomes and questionnaire data, as presented in Table 4.

**Table 4:** Study assessments

Item	Baseline	3 months	12 months (study end)
*Swedevox Register*	X		
Demographics	X		
Height and weight	X		
Spirometry	X		
Blood gases on air and oxygen	X		
Primary and secondary causes of starting LTOT	X		
Oxygen dose, equipment and duration	X		
Use of long-term mechanical ventilator	X		X
Date and reason of LTOT withdrawal		X	X
*Cause of Death Register*			
Date, place and causes of death		X	X
*Patient Register*			
Diagnoses/comorbidity and procedures	X	X	X
Hospitalizations	X	X	X
*Prescribed Drug Register*			
Dispensed drug prescription	X	X	X
*Clinical visits*			
Adverse events		(X)^a^	(X)^a^
*Patient questionnaire*			
Self-reported oxygen utilization		X	X
Breathlessness (MDP, NRS of worst, refractory, mMRC)		X	X
Fatigue (FACIT-Fatigue)		X	X
Self-reported physical activity (modified Grimby questionnaire)		X	X
Cognitive questionnaires (BAS, IQCODE and FAQ)		X	X
HRQoL (EQ -5D-5 L and CAT)		X	X
Global impression of change		X	X
Treatment preference		X	X

^a^Adverse events will be collected at the patient’s ordinary clinical visits. Abbreviations: *LTOT* long-term oxygen therapy, *MDP* Multidimensional Dyspnea Profile, *NRS* numeric rating scale, *mMRC* modified Medical Research Council, *FACIT* Functional Assessment of Chronic Illness Therapy, *IQCODE* Informant Questionnaire on Cognitive Decline in the Elderly, *BAS* Brief Anosognosia Scale *FAQ* Functional Activities Questionnaire, *HRQoL* Health Related Quality of Life, *CAT* the COPD Assessment Test, EQ-5D-5 L = the EuroQoL 5 dimensions - 5 levels

### Registry-based outcome assessments

The main endpoints of mortality and hospitalizations will be assessed using national Swedish governmental registries: date and causes of death (Causes of Death Register), and the date and diagnoses/procedural codes of hospitalizations (National Patient Register of in- and outpatient care) [20, 21]. Data will be cross-linked between registers using each participant’s unique Swedish identity number. In addition, data will be obtained on dispensed outpatient medications (Prescribed Drug Register) [22]. Endpoints will be assessed at three months and 12 months after randomization (study end). The completeness of the outcome registers ensures minimal loss of data and unbiased analysis according to the intention to treat principle.

### Patient questionnaires

At 3 months (range 2 to 5 months) and 12 months (range 10 to 14 months) after randomization, all non-deceased participants (according to the Population Register) will be sent a letter marked by the patient’s randomization number. This will be sent by the UCR Registry Unit (separated from the Clinical Research Unit responsible for managing of the study) and will include a standardized reminder about the allocated treatment to the patient and caregiver, a questionnaire including data on smoking and secondary endpoints, and instructions about how to fill out and return the questionnaires within 2 weeks in a stamped return envelope. If the questionnaire has not been returned to the UCR data management unit within 3 weeks, a reminder letter will be sent home to the patient. The questionnaire data will be entered into a database as reported (missing and incomplete data accepted) and quality checked by the UCR data management unit.

The questionnaire data will include the Multidimensional Dyspnea Profile (MDP) [23], the modified Medical Research Council (mMRC) breathlessness scale [24, 25], the Functional Assessment of Chronic Illness Therapy (FACIT)- fatigue scale [26], the Informant Questionnaire on Cognitive Decline in the Elderly (IQCODE) [27], the Brief Anosognosia Scale (BAS) [28], the Functional Activities Questionnaire (FAQ) [29], the COPD Assessment Test (CAT) [30], the Euro-QoL-5- dimensions-five levels (EQ-5D-5L) [31] and the modified Grimby activity questionnaire [32]. In addition, the worst breathlessness in the previous week will be self-rated on a numeric rating scale from 0 (neutral) to 10 (excruciating), and refractory breathlessness will be reported through answering yes to the question “Do you have breathlessness at rest or with minimal activity that is distressful? [33] The global impression of dyspnea scale will be used to provide information on the participant’s perception of their change in dyspnea since the start of the study, on a 7-point scale. Finally, the treatment reference of two allocation arms will be reported. The time for completion of the questionnaire is expected to be maximum an hour.

### Adverse events

Adverse events (AEs) will be collected at the patient’s ordinary clinical visits and documented. The investigators will assess the intensity and causality of the AE. Known side effects from oxygen treatment (e.g. nose and upper airways dryness and congestion) will therefore not be reported.

Events with the outcome death or hospitalization will not be reported as AE. These are primary and secondary endpoints which will be captured as registry data. For the same reason, incident diagnosis of cardiovascular disease will not be reported as AE.

### Quality assurance and control plans

The trial will be monitored in accordance with the principles of Good Clinical Practice (GCP). Both centralized monitoring activities and on-site monitoring activities will be used in this study. Based on a study design with very few study specific procedures, it has been decided that the major part of the monitoring will be performed using centralized methods.

The monitor will review source documents for verification of consistency with the study data base according to risk based monitoring. Data on the primary endpoint of mortality and of hospitalization will be complete due to follow-up using national registries. In terms of review of completed informed consent, a random sample of the patients will be checked by the monitor in connection with on-site visit. Sites will also log all completed informed consents and fax this log to the monitor on an on-going basis. The monitor will review the log to verify consistency with the study data base. If it is indicated that the site does not comply with the specified consent process, extended site-specific training and/or increased monitoring activities will be performed.

### Data management plan

The data management work will be performed by UCR. The Data Management Plan (DMP) describes the data flow within the study. A study database with all included patients will be generated, and the study specific randomization module and study specific variables from separate pCRFs (=Patient Questionnaire) will be entered into an Electronic Data Capture (EDC) system. Quality control procedures will apply and be described in the DMP, e.g. for entry of the patient questionnaires. All data will be merged at study end and exported to an analysis database for further analysis.

### Analysis plan

The primary analysis will be according to the intention to treat (ITT) principle and complemented with a per protocol (PP) analysis, as non-compliance to the allocated treatment may cause a potential bias towards equivalence between treatments. The primary endpoint of mortality will be analyzed with Cox’s proportional hazards model. Hospitalizations will be analyzed using Fine Gray regression, accounting for death as a competing event. Other secondary endpoints will be analyzed using two-sided Student’s t-tests for continuous variables (including HrQoL, breathlessness, cognition, fatigue, and activity scores, as well as health care usage) and chi-2 test for categorical variables (causes of death and patient’s preference of continuing LTOT). Correlational analyses, including of factors predictive of the primary and secondary outcomes, will be conducted using linear regression (continuous outcomes), logistic regression (categorical outcomes), and Cox and Fine-Gray regression models (time to event outcomes).

### Ethics, consent and permissions

A steering committee including all coauthors of the protocol paper are responsible for design, methodology and protocol amendments of the study. The study will be conducted in accordance with the protocol, applicable regulatory requirements such as, but not limited to, LVFS 2011:19, Good Clinical Practice and the ethical principles of the Declaration of Helsinki as adopted by the 18th World Medical Assembly in Helsinki, Finland, in 1964 and subsequent versions. The study (Protocol version 1.5, dated 2016-09-13) is approved by the Ethical Board of Lund, Sweden (DNr 2016/190) and by the Swedish Medical Products Agency (DNr: 5.1–2016-71,695). Recruitment started in May 2018.

## Discussion

This is the first study to compare effectiveness of LTOT duration of 24 and 15 h/day on clinical outcomes in people with chronic respiratory failure, with a potential direct impact on research and clinical management. LTOT is used commonly as it is one of few interventions that can affect prognosis in COPD, but there has been no trial of LTOT in severe hypoxemia since the 1970s [3, 4]. Research in LTOT has been held back by problems and high cost of recruitment and follow-up of patients with advanced disease, and challenges with ethical justification to withhold oxygen 24 h/day in patients with severe hypoxemia for the purpose of conducting a clinical trial even though there is no clear evidence of difference in outcomes between shorter and longer duration LTOT. This study will be by far the largest study in LTOT to date, and the first R-RCT in respiratory medicine. The R-RCT design is a paradigm shift and enables a large-scale, randomized trial in a representative sample of people with very advanced disease, with complete follow-up of main endpoints. This will be the first trial investigating whether LTOT 24 h/day improves important patient outcomes − survival time, risk of hospitalization, levels of symptoms, oxygen side effects, and HrQoL compared with LTOT 15 h/day, or whether LTOT 24 h/day might unnecessarily constraint and burden patients. This study will also for the first time evaluate effects of LTOT by the degree of hypoxaemia and in people with disease other than COPD. Further, evaluating the effect on the risk of hospitalization is crucial as hospitalization is a major driver of health care costs [34]. If COPD hospitalizations can be reduced by LTOT 24 h/day compared with 15 h/day, increased use of long term oxygen treatment could be cost-effective, which will be evaluated in a comprehensive health economic analysis. The trial will also examine the effect of LTOT prescribed 24 h/day compared with 15 h/day on survival and other outcomes, several of which were not assessed in the LOTT trial of patients with moderate hypoxemia [7]. The study will investigate effectiveness of the prescription of LTOT in clinical care. No objective measurement of oxygen use will be used, as there is no accepted, user-friendly objective means of collecting usage data. However self-reported actual oxygen duration will be collected.

If LTOT 24 h/day is found to be non-superior to 15 h/day, this will confirm that patients safely can be free of supplemental oxygen for up to nine hours per day. If not, LTOT 24 h/day may be superior which implies the importance of oxygen therapy in chronic respiratory failure and the relevance of implementing services to facilitate and optimize the daily duration of LTOT. The trial design will provide robust, detailed data using validated instruments that enable correlational analyses of factors influencing the adherence to and net clinical effectiveness of LTOT in patients with respiratory failure in current clinical practice. The study also aims to take forward an infrastructure for randomized trials that can be used to facilitate research and improved evidence-based treatment in patients with chronic respiratory failure.
